# New and resurrected Hawaiian species of pilo (*Coprosma*, Rubiaceae) from the island of Maui

**DOI:** 10.3897/phytokeys.60.6465

**Published:** 2016-02-11

**Authors:** Jason T. Cantley, Margaret J. Sporck-Koehler, Marian M. Chau

**Affiliations:** 1 Department of Biology, Bucknell University, 1 Dent Drive, Lewisburg, Pennsylvania 17837; 2 State of Hawai‘i, Department of Land and Natural Resources, Division of Forestry and Wildlife, 1151 Punchbowl Street, Honolulu, Hawai‘i 96813; 3 Lyon Arboretum Hawaiian Rare Plant Program, University of Hawai‘i at Mānoa, 3860 Mānoa Road, Honolulu, Hawai‘i 96822; 4 Department of Botany, University of Hawai‘i at Mānoa, 3190 Maile Way, Honolulu, Hawai‘i 96822

**Keywords:** Auwahi, Coprosma, Coprosma cordicarpa, Coprosma stephanocarpa, East Maui, Haleakalā, Hawaiian Islands, Kanaio, Maui, pilo, Rubiaceae, West Maui

## Abstract

Two species of *Coprosma* (Rubiaceae) J.R.Forst. & G.Forst. are described from the island of Maui of the Hawaiian Archipelago. A newly described taxon, *Coprosma cordicarpa* J.Cantley, Sporck-Koehler, & M.Chau, **sp. nov.** is locally common in medium to high elevation dry forests and shrublands of leeward East Maui. The second taxon is resurrected from the synonymy of *Coprosma foliosa* A.Gray as *Coprosma stephanocarpa* Hillebr. and occurs in mesic to wet rainforests of both East and West Maui. Both taxa are segregated from *Coprosma foliosa*, with which they share similar morphological characters. A conspicuous and persistent calyx of the fruit and various floral characters most easily differentiate both taxa from other Hawaiian taxa. The newly described *Coprosma cordicarpa* is further distinguished from *Coprosma stephanocarpa* by a central constriction of the fruit with a depressed apex, which gives it a characteristic heart shape. Furthermore, the taxa are largely separated phenologically, ecologically, and geographically. Descriptions, conservation status, and specimens examined for the new species are included.

## Introduction

There are more than 110 species in the genus *Coprosma* J.R. Forst. & G.Forst., which consists of species that are predominantly dioecious and wind pollinated. Species range in habit from trailing woody plants to large trees and produce many various colored fruits (e.g. black, blue, orange, red, yellow, and translucent), which are most often two-seeded drupes. The genus is Oceanic in distribution with a primary center of diversity in New Zealand (ca. 55 spp.: Glenny et al. 2010), and secondary centers of diversity in the Hawaiian Islands (13 spp.: Wagner et al. 1999), New Guinea (15 spp.: Garner 2002), Australia (8 spp.: Thompson 2010) and the Marquesas Islands (6 spp.: Wagner and Lorence 2011). Elsewhere, species are scattered widely across many islands and archipelagos of the Pacific Ocean from Borneo to the Juan Fernández Islands, but each island or archipelago has only one or two endemic species.

Cantley et al. (2014) indicate from their molecular phylogeny that the Hawaiian Islands were colonized by *Coprosma* during two independent colonization events: once for the black-fruited *Coprosma ernodeoides*, and secondly for all orange-fruited species. The orange-fruited Hawaiian *Coprosma* taxa—known locally as pilo in the Hawaiian language—were determined to be most closely related to the six Marquesan species and one (of two) species from Rapa Iti of the Austral Islands. Together, these taxa (from the Hawaiian Islands, Marquesas Islands, and Rapa Iti) were found to be more closely related to taxa in New Zealand than to other species on islands elsewhere in the Pacific where *Coprosma* taxa occur (i.e. Austral Is., Cook Is., Fiji, Kermadec Is., Lord Howe I., Norfolk I., Pitcairn I., Samoa, Society Is., and Vanuatu). Cantley et al. (2014) also determined that Hawaiian taxa were not closely related to Australian or Malesian taxa. Within the Hawaiian Islands, *Coprosma* taxa occur on all major islands except for Ni‘ihau and Kaho‘olawe. All taxa are endemic to the archipelago, but some maintain distributions across multiple islands. The intra-archipelago relationships among these taxa are not known. No resolution among Hawaiian taxa was recovered by Cantley et al. (2014), which they suggest is because the colonization event to the Hawaiian Islands by the orange-fruited *Coprosma* ancestor occurred after the emergence of Kaua‘i (≈5 Ma). Therefore, few detectable genetic mutations have since accumulated, which made it difficult to resolve recently diverged evolutionary relationships from the methodology that was used.

Species belonging to the genus *Coprosma* were first formally described in the Hawaiian Islands by Asa Gray in 1858 from material gathered by Nelson on Cook’s last voyage to the islands, and by Menzies during Vancouver’s voyage (Gray 1858). The descriptions of the taxa were brief, but included seven from the Hawaiian Islands. Following this, a number of individuals described additional taxa including: Wawra (1872), Hillebrand (1888), Heller (1896), Léveillé (1911), Rock (1913), and Oliver (1935, 1942). No new species of *Coprosma* have been described from the Hawaiian Islands since Oliver (1942). Only one comprehensive monograph of the genus was published (Oliver 1935), and therein was provided a thorough discussion of Hawaiian taxa, which highlighted Oliver’s dissatisfaction with attempting to fully delineate them with limited material, and without field observations. The most recent taxonomic treatment including all Hawaiian *Coprosma* taxa was by Wagner et al. (1999). In this treatment, six species and all varieties were sunk into synonymy. The resulting treatment details 13 endemic Hawaiian species in total from what was previously more than 20 taxa. Many taxa in Wagner (1999) are described as having a wide range of morphological diversity—such as for *Coprosma foliosa* A.Gray, *Coprosma ochracea* W. Oliver, and *Coprosma pubens* A.Gray. These species occur across multiple islands, whereas single island endemic taxa have much better defined morphologies (ex. *Coprosma ellpitica* W. Oliver of Kaua‘i or *Coprosma longifolia* A. Gray of O‘ahu). In some cases, Wagner et al. (1999) note at the end of the taxonomic descriptions that various Hawaiian *Coprosma* taxa are in need of fieldwork in order to better understand precise relationships and probable segregate taxa. In the field, Hawaiian *Coprosma* are notoriously difficult to quickly distinguish from one another as diagnostic characters among taxa are minute, and boundaries among species are not always clearly defined. Further complicating proper identification is that taxa are often found growing sympatrically, and are thought to hybridize occasionally. Without knowledge of variant morphologies of populations on different islands, it is often difficult to accurately identify a particular taxon in the field, even with the most current key to species (J. Cantley, pers. obs.).

Such is the case for currently described *Coprosma foliosa*, which is a widespread taxon occurring on islands of Kaua‘i, O‘ahu, Moloka‘i, Maui, and Lana‘i (Wagner 1999). It is effectively replaced on Hawai‘i Island by the morphologically similar *Coprosma menziesii* A.Gray. *Coprosma foliosa* was first described by Gray (1858), as a shrub with glabrous lanceolate to oblanceolate leaves and obovate to globose fruit with a naked apex. Following this, Hillebrand (1888) described a similar species, *Coprosma stephanocarpa* Hillebr. from Moloka‘i, Maui, and Kaua‘i, indicating the species had—as the name suggests—a fruit with a persistent calyx on the apex that is connate, forming a crown-like structure. The Kaua‘i specimens considered to represent *Coprosma stephanocarpa* were recognized by Gray (1858) as Coprosma pubens A.Gray var. kauensis A.Gray, and later were elevated to species level by Heller (1896). Hillebrand (1888) indicated the fruit of *Coprosma stephanocarpa* was globose *or* obovate with a depressed apex becoming bisulcate, and crowned by the spreading discreet calyx lobes. Rock (1913) then described *Coprosma vontempskyi* Rock from rainforests above Olinda, Maui, which shared similar morphological characters of Hillebrand’s *Coprosma stephanocarpa*, yet he made no mention of *Coprosma stephanocarpa* in his description. Oliver (1935) recognized this oversight by Rock and sank *Coprosma vontempskyi* into *Coprosma stephanocarpa* as he deemed them indistinguishable. Oliver revised the description of *Coprosma stephanocarpa* and restricted the fruit morphology to a “drupe [that is] ovoid…[and] crowned by the persistent calyx, 5-6 mm long.” However, the new restricted description of *Coprosma stephanocarpa* failed to the mention Hillebrand’s fruit characters describing the fruit as sometimes “obovate with a depressed apex, becoming bisulcate.” The bisulcate character was never again noted in further Hawaiian *Coprosma* taxonomic descriptions, including in Wagner et al. (1999) where *Coprosma stephanocarpa* (plus *Coprosma fauriei* H. Lév. and *Coprosma skottsbergiana* W. Oliver) was officially lumped into *Coprosma foliosa*. Oliver (1935) notes that his descriptions of *Coprosma stephanocarpa* were not fully satisfying to him as he felt unable to disentangle the diverse series, subspecies, forms, and potential hybrids of *Coprosma stephanocarpa*, particularly on East Maui, with only limited material, which was provided to him from his collaborator Harold St. John at the Bishop Museum in Honolulu.

The (re)discovery of bisulcate fruit with a depressed apex and a persistent crown-like calyx from a locally common *Coprosma* taxon in the Kanaio Natural Area Reserve on leeward East Maui, plus the difficulty in keying these individuals to the species level, prompted this detailed investigation by the authors. The investigation included in-depth herbarium research, as well as fieldwork to validate findings and to assess the taxon’s abundance and distribution on Maui. This paper recognizes one new distinct species, *Coprosma cordicarpa*, and unexpectedly confirms the need to resurrect *Coprosma stephanocarpa* from synonymy. Both taxa are segregated from *Coprosma foliosa* using morphological, ecological, phenological, and geographical lines of evidence. This study, plus a concurrent study of a new taxon from Kaua‘i (D. Lorence, pers. comm.), increases the total number of endemic *Coprosma* species in the Hawaiian Islands to 16.

## Methods

All measurements given herein are taken from dried herbarium specimens. Field observations were performed in September 2013, September 2014, and May 2015 to assess abundance and to take field notes and digital photos. Seeds of *Coprosma cordicarpa* were collected from two populations at Kanaio Natural Area Reserve and Auwahi totaling 609 seeds from 32 individual plants. All seeds were deposited for long-term germplasm storage at the Seed Conservation Laboratory at Lyon Arboretum. Measurements are presented in the descriptions as follows: length, then width, each followed by units of measurement (mm or cm). More than 80 specimens (including type specimens) from the BISH herbarium were studied and measured. Validations were also garnered from PTBG specimens. The area of occupancy (distribution) for each species was calculated using herbarium collection data and field observations. The conservation status is proposed following the IUCN Red List Category criteria (IUCN 2001; www.iucnredlist.org/info/categories_criteria2001). A file including measurements and notes taken from herbaria specimens is provided as supplemental data (See Suppl. material 1: *Coprosma* Morphology Data Matrix).

## Taxonomic treatment

### Coprosma

Taxon classificationPlantaeGentianalesRubiaceae

J.R. Forster & G. Forster

Coprosma Lectotype species (designated by Rehder 1949, pg. 597): *Coprosma foetidissima* J.R.Forst. & G.Forst.

#### Description.

Shrubs, multi-branched, erect, occasionally creeping and sometimes rooting at the nodes, or occasionally trees, often foetid when bruised. Leaves simple, opposite or rarely ternate, margins entire, petiolate or sessile; stipules interpetiolar, distinct or partly connate, entire or dentate with tooth-like marginal colleters. Flowers unisexual (and the plants dioecious or rarely monoecious), rarely polygamous or in one species perfect, axillary, solitary or in cymes; calyx 4–5(–10)-toothed, often reduced or absent in male flowers; corolla funnelform or campanulate, 4–5(–10)-lobed, lobes valvate in bud; stamens 4–5(–10), inserted at base of corolla tube; filaments long-exserted, erect or pendulous; ovary 2(–4)-celled, ovule 1 per cell, basal, anatropous; style 2(–4)-lobed, divided nearly to base; stigmas long-exserted, papillose-hirsute. Fruits drupaceous, fleshy, ovoid to globose, with 2(–4), 1-seeded, plano-convex pyrenes.

#### Key to the Hawaiian species of *Coprosma* previously treated as *Coprosma foliosa* s.l.

**Table:** 

1	Fruit reddish-orange, ovoid, lacking a central constriction between the two seeds, apex not depressed, calyx conspicuous or not	(**2**)
–	Fruit reddish-orange to yellow, cordate (heart-shaped) with a central constriction between the two seeds, apex depressed, calyx conspicuous and persistent, leeward East Maui	***Coprosma cordicarpa***
2	Fruit reddish-orange to yellow, fruit calyx conspicuous and persistent and >1.5 mm long, East and West Maui	***Coprosma stephanocarpa***
–	Fruit reddish-orange to yellow, persistent fruit calyx not present or sometimes minute (<1.5 mm), Kaua‘i, O‘ahu, Moloka‘i, Lana‘i, West Maui	***Coprosma foliosa* s.l.**

### Coprosma cordicarpa

Taxon classificationPlantaeGentianalesRubiaceae

J.Cantley, Sporck-Koehler, & M.Chau sp. nov.

urn:lsid:ipni.org:names:77152890-1

**Figure 1.**
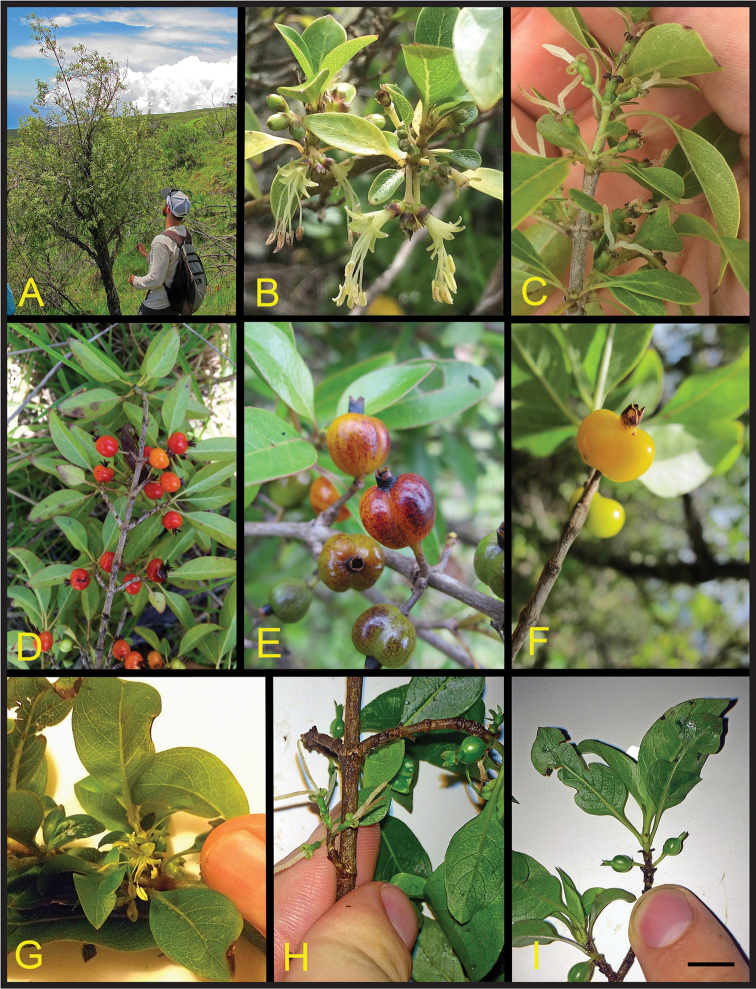
Field images of *Coprosma cordicarpa* and *Coprosma stephanocarpa*. **A–F** *Coprosma cordicarpa*. **A** habit and habitat of whole plant with JTC **B** male stem and inflorescences **C** female stem and inflorescences **D–F** fruits illustrating population variation in color and degree of calyx connation **G–H** *Coprosma stephanocarpa* **G** male stem and inflorescence **H** female stem and inflorescences **I** immature fruits. All images were taken by the authors, **A–C, E** from Kanaio Natural Area Reserve **D, F** from Auwahi **G–I** from Makawao Forest Reserve. Black scale bar at bottom right indicates the following lengths: 0.5 m (**A**), 1.5 cm (**B–C, E–F**), 5 cm (**D**), 2 cm (**G–I**).

**Figure 2.**
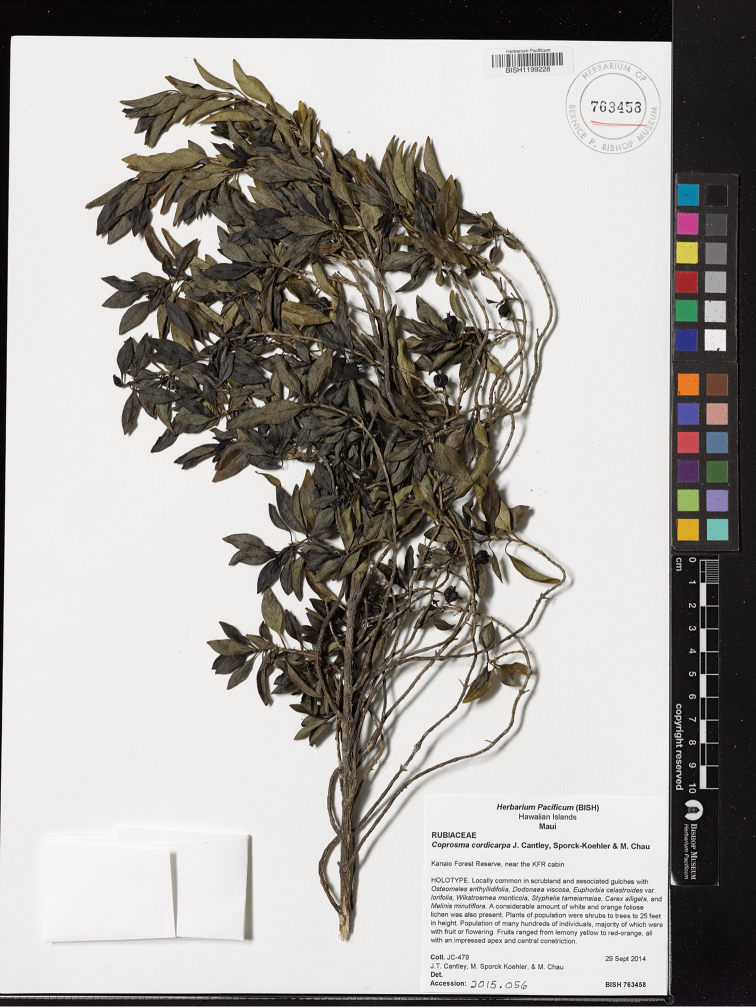
Photo of the holotype specimen of *Coprosma cordicarpa* (BISH).

Figure 1A–F ; Figure 2

#### Type.

**USA.** Hawai‘i: Maui: East Maui: Kanaio Natural Area Reserve, near cabin, 29 Sep 2014, *J.T. Cantley, M.J. Sporck-Koehler & M.M. Chau JC-479* (holotype: BISH 763458).

#### Diagnosis.

Differs from other currently recognized species of *Coprosma* in the Hawaiian Islands primarily by its cordiform fruits formed by a depressed apex and central constriction on female plants, and by large calyces (lobes 2–4 mm vs., for example, 0.25–2.00 mm of *Coprosma stephanocarpa* and *Coprosma foliosa* s.l.) on male flowers.

#### Description.

*Shrubs to trees* 2–7 m tall, with one to many main stems; young stems sparsely pubescent to puberulent; *Seedlings* and *Juveniles* with significantly more trichomes than mature individuals; *Leaves* opposite, blades 20–56 × 8–25 mm, lanceolate, both surfaces sparsely pilose or glabrous, midrib sparsely puberulent towards base, domatia present on abaxial surface in secondary vein axils, blade apex acute or sometimes rounded, base cuneate; petioles 5–12 mm long; stipules deltate 2–3 mm long, connate 25–50% of their length, base puberulent to lanate with band of glabrous tissue immediately below margin, margin lanate with a conspicuous apical colleter sometimes obscured by two marginal colleters. *Inflorescences* axillary; male inflorescences a 3-flowered cyme on an unbranched peduncle (3–)7–9(–17) mm long or sometimes trichotomously branched at base, with flowers terminal in groups of 3 on each branch, internodes 5–25 mm long, central branch up to twice as long; female inflorescence solitary, 3–7(–13) mm long or sometimes trichotomously branched at base with solitary flowers terminally occurring on lateral branches and central branch a 3-flowered cyme with sessile central flower, and two lateral flowers on short pedicels, internodes 5–25 mm long, central branch up to twice as long. *Flowers*: male calyx irregularly toothed, urceolate to campanulate in early development, becoming deeply split due to corolla growth, 2–4 mm long, sheathing basal 1/8–1/4 of the mature corolla, apex red-purple at maturity, corolla 5–6(–8)-merous, campanulate to widely funnelform, lobes 3 × 0.5 mm, stamens 5–6, inserted at base of corolla, filaments exserted to 7 mm, pendulous at maturity; female calyx irregularly toothed at margin, completely connate or nearly so, forming a cylindrical tube around the corolla or occasionally only 1/4 connate, 1.5–3.5 mm long, corolla 5–6-merous, narrowly funnelform to tubular, only lobes exerted beyond calyx, recurving 360 degrees at maturity, lobe apices often touching upper calyx near teeth, styles 2, divided to base, 3 cm long, ca. 0.5 mm diam. *Fruit* reddish-orange to lemony-yellow, sometimes with red to reddish-purple colored epidermal flecks, cordiform, tapering towards the base, 7–10 × 5–7 mm when dry, with central constriction (furrow) from base to apex present between two seeds, apex depressed between the two seeds, crowned with a conspicuous persistent calyx, drying brown. *Seeds* 2(–3) plano-convex pyrenes, yellowish-white, 2.3–6.3 × 2.5–5.0 mm × 1.0–3.0 mm, seed operculum 0.5–2.1 mm long.

#### Phenology.

Flowering specimens were collected from August to September except for one individual in March. Field observations of September 2013 and 2014 found that most individuals of the population at Kanaio Natural Area Reserve and Auwahi were fruiting, and only few flowering. Fruiting specimens were collected across many months, but it is unknown how long fruits were mature on individuals prior to collection.

#### Distribution.

Known only from southern, leeward slopes of East Maui (Haleakalā) at elevations of approximately 1000-2000 m, roughly spanning east to west from the Kanaio Natural Area Reserve to the Kaupō Gap Trail. The linear distance is estimated at approximately 21 km, but populations may be disjunct, especially in poor, degraded habitat where ungulates and invasive plant species (i.e. *Cenchrus clandestinus* (Hochst. ex Choiv.) Morrone) are dominant. Known locations include Kanaio Natural Area Reserve, Auwahi, Kahikinui Forest Reserve, Nu‘u, and the Kaupō Gap Trail. Modern observations of abundance (2013-2015) at Kanaio Natural Area Reserve, Auwahi and Kaupō Gap Trail indicate that it is locally common at all sites. Its present abundance in Nu‘u and Kahikinui Forest Reserve is not known.

#### Ecology.

In native habitats, *Coprosma cordicarpa* occurs in dry forest/shrubland habitat with *Chrysodracon auwahiensis* (H. St. John) P.L.Lu & Morden, *Dodonaea viscosa* Jacq., Euphorbia celastroides (Boiss) Croizat & Degener var. lorifolia A.Gray, *Osteomeles anthyllidifolia* (Sm.) Lindl., *Leptecophylla tameiameiae* (Cham. & Schltdl.) C.M.Weiller, and an understory of *Carex wahuensis* C.A.Mey. It is often present in invaded habitats with *Cenchrus clandestinus*. It occurs primarily in open habitat receiving direct sunlight, but was observed in gulches and high elevation forests along the Kaupō Gap Trail (Seana Walsh, pers. comm.). The rainfall in the distribution of *Coprosma cordicarpa* varies from 700 to 1900 mm annually with the highest rainfall occurring from December to January (Giambelluca et al. 2013). Flowering occurs during the dry season, and fruits appear to mature shortly preceding the wettest months, which may represent a germination strategy for this dry habitat taxon.

#### Etymology.

The specific epithet refers to the heart-shaped fruit, which is a product of the central constriction of the fruit and depressed apex. This character is unique among Hawaiian *Coprosma* taxa.

#### Conservation status.

This taxon occurs as scattered individuals that are locally common within five populations on one volcano. When evaluated using the IUCN criteria for extinction risk (IUCN 2012), *Coprosma cordicarpa* falls into the Vulnerable (VU) category under Criterion B1ab(iii)+2ab(iii). The VU designation is the lowest of three threatened categories, but indicates the taxon still faces a high risk of extinction in the wild. It has an area of occupancy < 2000 km^2^ and extent of occurrence < 20,000 km^2^, less than 10 known locations that are possibly fragmented, and an observed continuing decline in habitat quality overall. Such suitable habitat may continue decreasing in size without active conservation management. In areas where the landscape has been actively managed for ungulates and invasive plant species (i.e. Auwahi, Kanaio Natural Area Reserve), *Coprosma cordicarpa* purportedly has displayed a marked natural increase in population size. Further conservation measures may lower extinction risk and change the threatened status of *Coprosma cordicarpa*.

#### Specimens examined.

**United States of America. Hawai‘i: Maui: East Maui**: Hana District, Kaupo Gap, ≈1.75 mi S of Paliku Cabin, alt. 5000 ft, 16 Jul 1969, *J.S. Henrickson 5000* (BISH); Haleakalā, Kaupo Gap, 2nd cove S of Waikeke'ehia, 6000 ft, 27 Jun 69, *H. St.John 21189* (BISH); Auwahi, 4000 ft, 30 Jun 72, *H. St. John 26860* (BISH); Auwahi District, S slope, 18 Dec 1981, *A.C. Medeiros 195* (BISH); Auwahi District, S slope, 18 Dec 1981, *A.C. Medeiros 195* (BISH); Auwahi, 0.5 km SE of Pu‘u O‘uli, alt. ca. 4000 ft, 24 Jun 1980, *P.K. Higashino 9254* (BISH); Auwahi, 24 Nov 1920, *C.N. Forbes 2096M* (BISH); Auwahi, 20 Mar 1920, *C.N. Forbes 2043M* (BISH); Auwahi, 1 Feb 1953, *J.F.C. Rock 27003* (BISH); 8 mi. E of Ulupalakua, S slope of Haleakalā, alt. 3000-3500 ft, 26 Aug 1976, *E.L. Little Jr. 31132* (BISH); SW slope of Haleakalā, Kahikinui FR, S of Kahua Cabin Rd, alt. ca. 4900 ft, 24 Jun 1980, *F.R. Warshauer 2684* (BISH); Kahikinui, S Haleakalā , 0.1-0.5 km W of Manawainui Gulch, alt. ca. 4840 ft, June 20, 1980, *P.K. Higashino 9234* (BISH); Kanaio NAR, 13 Sept 2013, *M.J. Sporck-Koehler s.n.* (BISH); Nui, S slope of Haleakalā, 6 Mar 1920, C.N. Forbes 1858M (BISH); Near Kanaio NAR Cabin, 29 Sep 2014, *J.T. Cantley, M.J. Sporck-Koehler & M.M. Chau JC-475* (BISH); Near Kanaio NAR Cabin, 29 Sep 2014, *J.T. Cantley, M.J. Sporck-Koehler & M.M. Chau, JC-476* (BISH); Near Kanaio NAR Cabin, 29 Sep 2014, *J.T. Cantley, M.J. Sporck-Koehler & M.M. Chau JC-477* (BISH); Near Kanaio NAR Cabin, 29 Sep 2014, *J.T. Cantley, M.J. Sporck-Koehler & M.M. Chau JC-478* (BISH); Near Kanaio NAR Cabin, 29 Sep 2014, *J.T. Cantley, M.J. Sporck-Koehler & M.M. Chau JC-479* (Holotype: BISH); Auwahi restoration unit #1, 29 Sep 2014, *J.T. Cantley, M.J. Sporck-Koehler & M.M. Chau JC-480* (BISH); Kahikinui, area east of Manawainui, alt. 5000-6000 ft, *K.R. Wood 6247* (PTBG).

### Coprosma stephanocarpa

Taxon classificationPlantaeGentianalesRubiaceae

Hillebr.

**Figure 3.**
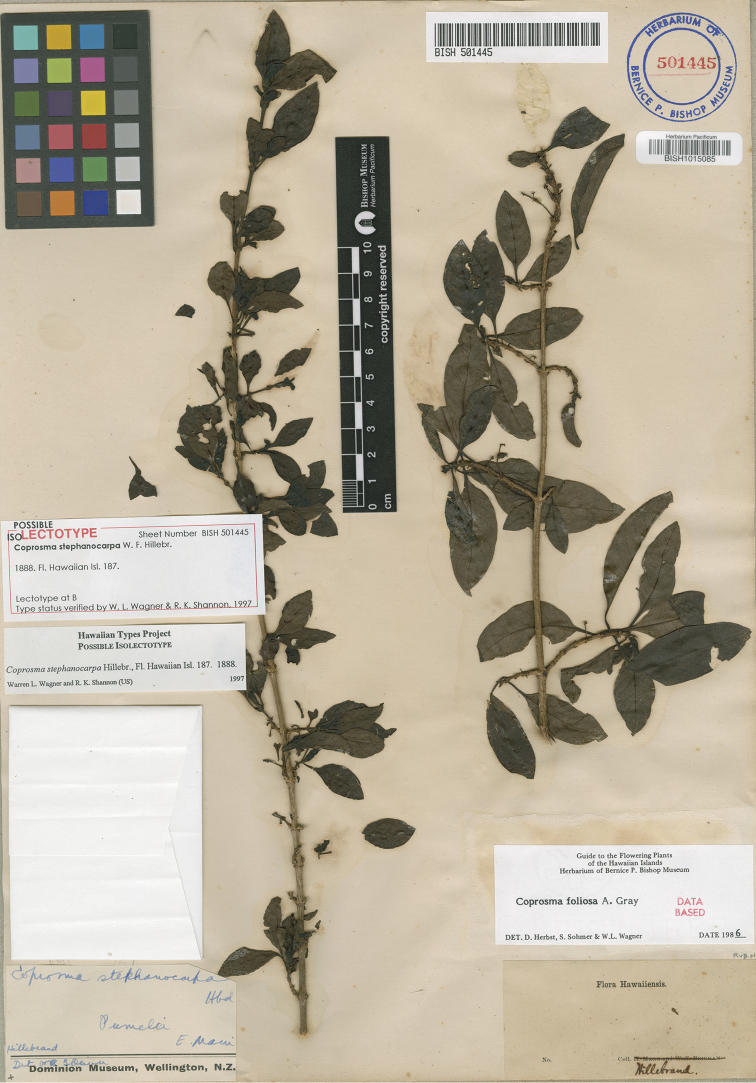
Photo of the holotype specimen of *Coprosma stephanocarpa* (BISH).

Figure 1G–I ; Figure 3

#### Type.

United States of America. Hawai‘i: Maui, East Maui, Haleakalā, alt. 3000-6000 ft, 1888, *J. Hillebrand* s.n. (lectotype, designated by Oliver 1935, pg. 164: isolectotype: BISH!)

#### Description.

*Shrubs to small trees* 2–6 m tall, with one to many main stems; young stems sparsely pubescent. *Leaves* opposite, blades 6–50 × 4–15 mm, elliptic or sometimes lanceolate, sparsely pilose on both surfaces, midrib puberulent, domatia present on abaxial surface in secondary vein axils, apex acute, base cuneate to attenuate, petioles 3–5(–10) mm long; stipules narrowly deltate, often recurving away from stem, 2–4 × 2–3 mm, connate 1/4 to 1/2 of their length, base puberulent to lanate, margins lanate with one apical colleter and no marginal colleters, band of glabrous tissue immediately below the margin. *Inflorescences* axillary, male inflorescence a 3(–5) flowered cyme on an unbranched peduncle 3–5 mm long; female inflorescence solitary or occasionally 2-3, sessile or subsessile, peduncle 0–3(–6) mm long, unbranched, flowers terminal. *Flowers* male calyx lobes 3–5, irregularly toothed 0.25-2 mm long, corolla 5–6-merous, funnelform, 3–4 mm long, lobes 0.5–1.5 mm long, tube 3–4 mm long beyond calyx, stamens 5–6, anthers inserted at base of corolla, filaments 7–13 mm long, exserted beyond corolla, pendulous at maturity; female calyx 3–5 lobes 0–2 mm long, corolla funnelform to campanulate, only lobes exserted beyond calyx, recurving 360 degrees at maturity, lobe apices often touching calyx, styles 2, divided to base, exserted 3–4 mm beyond corolla. *Fruit* reddish-orange to yellow, ovoid, 4–10 × 3.5–6 mm, crowned with a conspicuous persistent calyx. *Seeds* 2 plano-convex pyrenes, yellowish-white.

#### Phenology.

Most flowering specimens were collected from December to February and a lesser number from July to August. Specimens from Lihau (West Maui) are only known to flower in July. No individuals were fruiting or flowering in late September 2014 in Makawao Forest Reserve, but immature fruits and flowers were observed in May 2015. Most fruiting specimens occurred in July, but collections were made across many months.

#### Distribution.

Known from East and West Maui, but apparently more prevalent on East Maui. The taxon occurs from approximately 975m to 1700m elevation. On East Maui, it is known from mesic sites from Kīpahulu Valley to Olinda. Collections on West Maui are collected from Lihau and Honokawai.

#### Ecology.

Found in mesic to wet forests and shrublands with both native and non-native plant communities. Occurs primarily as an understory shrub to small tree. The rainfall varies dramatically across its distribution and precise collection localities should be geo-referenced to provide an accurate range of precipitation requirements for this taxon’s distribution.

#### Etymology.

The specific epithet refers to the persistent calyx on the fruit apex that looks like a crown (crown in Greek = stephanos) due to its persistence, connation, and irregular dentations.

**Conservation status.** This taxon occurs as scattered individuals that are locally common on two volcanoes within one island. When evaluated using the IUCN criteria for extinction risk (IUCN 2012), *Coprosma stephanocarpa* falls into the Vulnerable (VU) category under Criterion B1ab(iii)+2ab(iii). The VU designation is the lowest of three threatened categories, but indicates the taxon still faces a high risk of extinction in the wild. It has an area of occupancy < 2000 km^2^ and extent of occurrence < 20,000 km^2^, less than 10 known locations, and an observed continuing decline in habitat quality overall. Suitable habitat may continue decreasing in size without active conservation management, and currently none of the populations occur in protected areas.

#### Specimens examined.

**United States of America. Hawaii: Maui: West Maui**: edges of Honokowai gulch, alt. 4500 ft, 24 Aug 1910, *J.F.C. Rock 8189* (BISH); Manawainui Gulch, alt. 820 m, 8 Mar 1988, *W.L. Wagner 5857* (BISH, PTBG); Lahaina District, Lihau, alt. 3200 ft, 28 Oct 1991, *P. Welton 1379* (BISH); Lahaina District, Lihau, alt. 3250 ft, 9 May 1991, *P. Welton 911* (BISH); Lahaina District, Lihau, alt. 3700 ft, 9 May 1991, *P. Welton 954* (BISH); Lahaina District, Lihau, alt. 3800 ft, 25 Aug 1991, *P. Welton 1237* (BISH); Lahaina District, Lihau, alt. 3800 ft, 26 Aug 1991, *P. Welton 1261* (BISH); Lahaina District, Lihau, alt. 3900 ft, 9 May 1991, *P. Welton 955* (BISH); Lahaina District, Lihau, alt. 4000 ft, 24 Aug 1991, *P. Welton 1177* (BISH); Lahaina District, Lihau, alt. 4000 ft, 24 Aug 1991, *P. Welton 1178* (BISH); Lihau, alt. 4100 ft, 13 Jul 1991, *P. Welton 1091* (BISH); Lahaina District, Lihau, alt. 4120 ft, 25 Aug 1991, *P. Welton 1200* (BISH); Kukui watershed area, ridge towards Kahana Valley between Kahana and Kahana Iki valleys, *M. Kiehn MK-990913-2/4* (PTBG). **East Maui**: Olinda Forest Reserve, 26 Mar 1952, *O. Degener 22116* (BISH); Olinda Forest Reserve, 26 Mar 1952, *O. Degener 22116* (BISH); Makawao, Olinda pipe line road, lower fork, alt. 4650 ft, 25 Dec 1951, *A.K. Chock 298* (BISH); Olinda FR, Waikamoi Flume, 12 Jan 1985, *L. Pyle & W. Takeuchi 1923* (BISH); Olinda FR, alt. 4025 ft, 12 Jan 1985, *L. Pyle & W. Takeuchi 1922* (BISH); Olinda, 24 Apr 1918, *G.C. Munro 482* (BISH); Olinda, 3 May 1967, *N.L.H. Krauss 1128* (BISH); 2 mi. E of Olinda, alt. 4200 ft, 27 Jun 1969, *J.S. Henrickson 3738* (BISH); Haleakalā, below Olinda, 24 Apr 1918, *G.C. Munro 648* (BISH); lower Olinda Flume, between gate and road, alt. ca. 4000 ft, 19 Jul 1964, *M.R. Crosby 1804* (BISH); above Olinda, alt. 4200 ft, 30 Sep 1945, *A.L. Mitchell 65* (BISH); Kula pipe line, Olinda, 4500 ft, 11 Feb 1930, *H. St. John 10296* (BISH); Kula Pipe Line, 25 May 1930, *O.H. Swezey s.n.* (BISH); Makawao Forest Reserve, Olinda pipe line, alt. 4300 ft, 25 Dec 1955, *H.A. Woolford 137* (BISH); Makawao, Olinda pipe line road, lower fork, alt. 4650 ft, 25 Dec 1951, *A.K. Chock 298* (BISH); Kula pipe line, 18 Oct 1922, *C.J.F. Skottsberg 892* (BISH); Makawao Forest Reserve, alt. 4300 ft, 16 Jul 1980, *K.T. Adee s.n.* (BISH); Makawao District, Kalialinui, NW Haleakala, Pu'u Luau, ca. 5720 ft, 10 Jun 1980, *P.K. Higashino 9340* (BISH); Ko'olau Forest Reserve, S of W Wailuaiki Stream on pali, alt. ca. 5310 ft, 31 May 1980, *P.K. Higashino 9047* (BISH); E of Ukulele, 17 Jul 1919, *C.N. Forbes 816M* (BISH); Ukulele, 1 Jul 1919, *C.N. Forbes 748M* (BISH); Ukulele, 1 Jul 1919, *C.N. Forbes 758M* (BISH); Ukulele, 1 Jul 1919, *C.N. Forbes 769M* (BISH); Ukulele, 1 Jul 1919, *C.N. Forbes 936M* (BISH); Ukulele, 1 Jul 1919, C.N. Forbes 748M (BISH); Ukulele, 1 Jul 1919, *C.N. Forbes 738M* (BISH); Ukulele, 1 Jul 1919, *C.N. Forbes 7 69M* (BISH); above Ukulele, 1 Jul 1910, *C.N. Forbes 215*M (BISH); Ukulele, *C.N. Forbes s.n.* (BISH); Pu‘u Pani, 4 Mar 1920, *C.N. Forbes 1836M* (BISH); Waikamoi, alt. 4250 ft, 14 Aug 1933, *M.C. Neal s.n.* (BISH); Makawao Forest Reserve, Waikamoi Flume Rd, 9 Jan 1997, *C.R. Annable 3231* (BISH); N slope of Haleakalā, between Hanawī and E fork of Kopiliula streams, alt. ca. 5750 ft, 13 Jul 1980, *P.K. Higashino 9375* (BISH); Hana, E of Kuhiwa Stream and Valley, ca. 5540 ft, 13 Jun 1980, *P.K. Higashino 9146* (BISH); Kīpahulu, L side of valley, 15 Nov 1919, *C.N. Forbes 1639M* (BISH); Kipahulu, ridge L side of valley, 15 Nov 1919, *C.N. Forbes 1648M* (BISH); Hana, ridge of central part of Kipahulu Valley, alt. 3780 ft, 22 Jul 1980, *P.K. Higashino 9407f* (BISH); Kipahulu Forest Reserve, ridge N of Pu‘u Ahuli‘i, alt. ca. 5400 ft, 23 Aug 1980, *F.R. Warshauer 3113* (BISH); 1-3 km E of Kaupo Gap, Kipahulu Forest Reserve, alt. ca. 5750 ft, 27 May 1980, *F.R. Warshauer 2560* (BISH).

#### Discussion.

When numerous collections of *Coprosma foliosa* from Maui were run through the most current key to Hawaiian *Coprosma* (Wagner et al. 1999), it was clear that at least two taxa subsumed under that species merited taxonomic recognition. Both taxa failed to key out, and specimens did not closely match the taxonomic description of *Coprosma foliosa* or any other species. After multiple field visits, herbarium specimen measurements, and an in-depth literature review, it was concluded that the two *Coprosma foliosa* segregates, *Coprosma cordicarpa* and *Coprosma stephanocarpa*, maintain rather consistent morphologies on leeward East Maui (*Coprosma cordicarpa*) and mesic areas of East and West Maui (*Coprosma stephanocarpa*). They can easily be segregated from the variable *Coprosma foliosa* s.l. found on other islands and effectively replace *Coprosma foliosa* s.l. on East Maui, although at least one form of *Coprosma foliosa* s.l. is still found on West Maui. *Coprosma cordicarpa* is most easily distinguished from *Coprosma foliosa* s.l. by its heart-shaped fruit characters, which include a depressed apex crowned by a persistent connate calyx and the tendency to become bisulcate, particularly when dry. Male individuals of *Coprosma cordicarpa* are easily differentiated as the calyx is nearly double in size (2-4 mm) than that of *Coprosma foliosa* s.l. (0.25-2 mm), and *Coprosma stephanocarpa* (0.25-2 mm). The male calyx ontogeny of *Coprosma cordicarpa* is quite striking; the irregularly toothed calyx initially appears to be completely connate (or nearly so) when in bud, but due to expansion of the growing corolla, the calyx is mechanically split (often in two locations) becoming deeply incised, and then brown callus tissue forms. Ecologically, *Coprosma cordicarpa* is found in a unique habitat niche (dry forest/shrubland) compared to *Coprosma foliosa* s.l. and *Coprosma stephanocarpa*. The niche requirements of *Coprosma cordicarpa* should be studied in more detail, but it is clear that the areas where it occurs receive much less precipitation than *Coprosma stephanocarpa* habitat, which include mesic to wet locations of East and West Maui.

Phenological observations suggest that *Coprosma cordicarpa* and *Coprosma stephanocarpa* have different flowering periods that are primarily non-overlapping. *Coprosma cordicarpa* has a primary flowering period in late summer (August to September). The flowering period of *Coprosma stephanocarpa* is primarily the winter (December to February), with a less pronounced period of flowering from July to August. Scant information about Hawaiian *Coprosma* phenology has been published, but *Coprosma ochracea* and *Coprosma rhynchocarpa* from Hawai‘i Island have a peak flowering period during early spring months (March to May; Lamoureux 1973), which is different from the summer flowering *Coprosma cordicarpa*, and winter flowering *Coprosma stephanocarpa*. Phenological differences among the evolution of Lord Howe Island species of *Coprosma* have proven to be evolutionarily significant (Papadopolos et al. 2011), and a similar case could also be true for Hawaiian taxa. However, it should be noted that while most, if not all, taxa of Hawaiian *Coprosma* have a primary robust flowering period each year, a small percentage of individuals in any population can occasionally be sporadically flowering across many different months (J. Cantley, pers. obs.). The stochasticity of flowering times could help explain the presence of occasional hybrids that are thought to exist among many sympatrically occurring *Coprosma* taxa in Hawai‘i.

The taxonomic boundaries between *Coprosma stephanocarpa* and *Coprosma foliosa* s.l. are less easily defined, especially from populations of *Coprosma foliosa* s.l. occurring on the islands of Moloka‘i and Lana‘i. However, *Coprosma stephanocarpa* is here restricted to Maui only, as taxa elsewhere appear to maintain consistent morphological differences. On Moloka‘i, at least two morphotypes of *Coprosma foliosa* s.l. exist that are similar to *Coprosma stephanocarpa*. One of these taxa has smaller fruits that are globose with a naked apex, smaller floral characters, and smaller leaves in general, but other vegetative characters (e.g. stipule size & pubescence) are similar to *Coprosma stephanocarpa*. The second Moloka‘i *Coprosma foliosa* s.l. morphotype has broad stipules that are more or less glabrous and ellipsoid to globose fruits with a small persistent crown that is not longer than 0.75 mm in length. Concerning Lana‘i specimens, few collections have been made, but these agree in morphology with the former described small-fruited, small-leaved Moloka‘i taxon, although much less morphological variation was noted. Fieldwork is needed on both islands to better understand these taxa and their relationship with *Coprosma stephanocarpa*.

#### Conclusions.

The recognition of *Coprosma cordicarpa* and *Coprosma stephanocarpa* brings the total number of *Coprosma* species described in the Hawaiian Islands to 15. A concurrent study of material collected on Kaua‘i will increase the total number of taxa described to 16 (D. Lorence, pers. comm.). Ongoing investigation of the *Coprosma foliosa* s.l. complex on Maui and other islands (Kaua‘i, O‘ahu, Moloka‘i, and Lana‘i) is currently being pursued by the authors. Preliminary investigation suggests that perhaps morphology is correlated with geographical location, which may support the need for resurrection of other currently synonymized or novel taxa not discussed in this paper. Moreover, detailed investigations of other currently valid taxa, such as the variable *Coprosma pubens* and *Coprosma ochracea*, could reveal cryptic taxa by understanding their diversity in better detail at the population level. Ultimately, it is suggested that a molecular study be undertaken to help shed light on the interesting evolutionary patterns of speciation for this dynamic genus in the Hawaiian Islands.

## Supplementary Material

XML Treatment for Coprosma

XML Treatment for Coprosma cordicarpa

XML Treatment for Coprosma stephanocarpa

## Supplementary materials

Supplementary material 1 *Coprosma* Morphology Data Matrix Data type: Measurement Explanation note: Measurements and notes taken from herbaria specimens. This dataset is made available under the Open Database License (http://opendatacommons.org/licenses/odbl/1.0/). The Open Database License (ODbL) is a license agreement intended to allow users to freely share, modify, and use this Dataset while maintaining this same freedom for others, provided that the original source and author(s) are credited.Jason T. Cantley, Margaret J. Sporck-Koehler, Marian M. Chau
